# 

**Published:** 1896-08

**Authors:** 


### CONTRIBUTORS TO VOLUME XXXVI.

| | |
|---|---|
| Angell, Edward B | Rochester. |
| Bissell, William G | Buffalo. |
| Busch, Frederick C | Buffalo. |
| Carpenter, Thomas B | Buffalo. |
| Cheesman, William S | Auburn. |
| Clark, Horace | Buffalo. |
| Clemesha, J. C | Buffalo. |
| Crockett, M. A | Buffalo. |
| Cumston, Charles Greene | Boston. |
| Dagenais, Alphonse | Buffalo. |
| Daggett, Byron H | Buffalo. |
| Diehl, Alfred E | Buffalo. |
| Dowd, J. Henry | Buffalo. |
| Dunham, Sidney A | Buffalo. |
| Ellis, Charles A | Buffalo. |
| Frye, Maud Josephine | Buffalo. |
| Gould, George M | Philadelphia. |
| Hauenstein, John | Buffalo. |
| Himmelsbach, George A | Buffalo. |
| Hinkel, Frank Whitehill | Buffalo. |
| Hubbell, Alvin A | Buffalo. |
| Hurd, Arthur W | Buffalo. |
| Jacobson, Nathan | Syracuse. |
| Keyes, William C | Buffalo. |
| Krauss, William C | Buffalo. |
| Loveland, B. C | Clifton Springs. |
| Mann, Matthew D | Buffalo. |
| McMurtry, Lewis S | Louisville. |
| Miller, John A | Buffalo. |
| Moehlau, F. G | Buffalo. |
| Moore, V. A | Ithaca. |
| O'Neill, J. M | Buffalo. |
| Park, Roswell | Buffalo. |
| Palmer, John O | Auburn. |
| Parmenter, John | Buffalo. |
| Potter, William Warren | Buffalo. |
| Pryor, John H | Buffalo. |
| Putnam, James O | Buffalo. |
| Putnam, James Wright | Buffalo. |
| Rafter, George W | Rochester. |
| Richmond, Charles H | Livonia Station. |
| Richmond, Nelson G | Fredonia. |
| Reed, Charles A. L | Cincinnati. |
| Rider, Wheelock | Rochester. |

Roe, John O . . . . . . . . . . . . . . . . . . . . . . . . . . . Rochester.
Roosa, D. B. St. John . . . . . . . . . . . . . . . . . . . . . . New York.
Rulison, E. T . . . . . . . . . . . . . . . . . . . . . . . . . . . Buffalo.
Satterlee, Richard H . . . . . . . . . . . . . . . . . . . . . . . Buffalo.
Sefton, Frederick . . . . . . . . . . . . . . . . . . . . . . . . . Auburn.
Snow, Irving M . . . . . . . . . . . . . . . . . . . . . . . . . . Buffalo.
Stanton, William . . . . . . . . . . . . . . . . . . . . . . . . . Varysburg.
Stephenson, F. H . . . . . . . . . . . . . . . . . . . . . . . . . Syracuse.
Stockton, Charles G . . . . . . . . . . . . . . . . . . . . . . . . Buffalo.
Sturtevant, J. R . . . . . . . . . . . . . . . . . . . . . . . . . Theresa.
Tait, Lawson . . . . . . . . . . . . . . . . . . . . . . . . . . . Birmingham.
Thompson, Justin G . . . . . . . . . . . . . . . . . . . . . . . . Angola.
Ullman, Julius . . . . . . . . . . . . . . . . . . . . . . . . . . Buffalo.
Wende, Ernest . . . . . . . . . . . . . . . . . . . . . . . . . . . Buffalo.
Wende, Grover William . . . . . . . . . . . . . . . . . . . . . . Buffalo.
Williams, H. T . . . . . . . . . . . . . . . . . . . . . . . . . . Rochester.
Young, A. A . . . . . . . . . . . . . . . . . . . . . . . . . . . . Newark.
American Association of Obstetricians and Gynecologists.
American Public Health Association.
Medical Association of Central New York.
Medical Society County of Chautauqua.
Medical Society County of Erie.

### BOOKS RECEIVED.

Transactions of the Medical Society of the State of New York for the Year 1896. Frederic C. Curtis, M. D., Secretary, Albany, N. Y. Philadelphia: Wm. J. Dornan, Printer, 100 N. Seventh street. 1896.

Keil's Medical, Pharmaceutical and Dental Register-Directory and Intelligencer. With special Medical, Pharmaceutical and Dental Departments, containing detailed information of colleges, hospitals, asylums, societies; with street lists, etc., etc., for Pennsylvania, New York, New

Jersey, Maryland, Delaware and District of Columbia. Fourth edition. George Keil, editor. Philadelphia: Burk & McFetridge Co., Publishers, 306 Chestnut street. 1896.

Transactions of the Southern Surgical and Gynecological Association. Eighth annual meeting, held at Washington, D. C., November 12, 13 and 14, 1896. W. E. B. Davis, M. D., Secretary, Birmingham, Ala. Published by the Association. Philadelphia: Wm. J. Dornan, Printer, 100 N. Seventh street. 1896.

Practical Points in Nursing, for Nurses in Private Practice. With an appendix containing rules for feeding the sick, recipes for invalid foods and beverages, weights and measures, dose list and a full glossary of medical terms and nursing treatment. By Emily A. M. Stoney, Graduate of the training school for nurses, Lawrence, Mass.; Superintendent of training school for nurses, Carney Hospital, South Boston, Mass. Octavo, pp. 456. Illustrated. Price, $1.75 net. Philadelphia: W. B. Saunders, 925 Walnut street. 1896.

A Manual of Obstetrics. By W. A. Newman Dorland, A. M., M. D., Assistant Demonstrator of Obstetrics, University of Pennsylvania; Instructor in Gynecology in the Philadelphia Polyclinic etc. Octavo, pp. 760. Illustrated. Price, $2.50 net. Philadelphia: W. B. Saunders, 925 Walnut street. 1896.

The Multum in Parvo Reference and Dose Book. By C. Henri Leonard, M. A., M. D., Professor of the Medical and Surgical Diseases of Women, Detroit College of Medicine. Flexible leather, 143 pages. Price, 75 cents. Detroit: The Illustrated Medical Journal Co., Publishers. 1896.

In Sickness and in Health. A Manual of Domestic Medicine and Surgery, Hygiene, Dietetics and Nursing. Dealing in a practical way with the problems relating to the maintenance of health, the prevention and treatment of disease and the most effective aid in emergencies. Edited by J. West Roosevelt, M. D., Late Physician in Charge of Seton Hospital for Consumptives; Visiting Physician to Bellevue Hospital, and Attending Physician to Roosevelt Hospital, New York. Complete in one volume of over a thousand pages. Illustrated with four colored plates and numerous engravings. Full analytical index. Sold only by subscription. Price, $6.00, in handsome cloth binding. Delivered to any address in the United States or Canada. New York: D. Appleton & Co., Publishers, 72 Fifth avenue. 1896.

Transactions of the American Association of Obstetricians and Gynecologists. Volume VIII. Eighth annual meeting, held at Chicago, September 24, 25 and 26, 1895. William Warren Potter, M. D., Secretary, Buffalo, N. Y. Philadelphia: Wm. J. Dornan, Printer, 100 N. Seventh street. 1896.

Anregung zur Reform der Physiologie des Menschen von Dr. F. Jezek. Mit Zahlreichen Abbildungen im Texte. Stuttgart: Hobbing & Büchle. 1896.

A System of Surgery. By American authors. Edited by Frederic S. Dennis, M. D., Professor of the Principles and Practice of Surgery, Bellevue Hospital Medical College, New York; President of the American Surgical Association, etc., assisted by John S. Billings, M. D., LL. D., D. C. L., Deputy Surgeon-General, U. S. A. Complete in four imperial octavo volumes, containing 3,652 pages, with index. 1,585 engravings and forty-five full-page plates in black and colors. Volume IV., 970 pages, 441 engravings and twenty-three plates. Price, per volume, $6.00 in cloth; $7.00 in leather; $8.50 in half Morocco, gilt back and top. For sale by subscription. Full circular free to any address on application to the publishers. Philadelphia: Lea Brothers & Co. 1896.

